# New evidence concerning the pathomechanism and treatment of thyroid associated orbitopathy

**DOI:** 10.1186/1756-6614-8-S1-A6

**Published:** 2015-06-22

**Authors:** Jacek Daroszewski

**Affiliations:** 1 Department of Endocrinology, Diabetes and Isotope Therapy, Wroclaw Medical University, Poland

## 

Thyroid associated orbitopathy (TAO) is an immune-mediated inflammatory disorder that causes expansion of the orbital adipose tissue and muscles and deposition of glycosaminoglycans and collagen. No specific therapy has been established, and treatment still relies on high-dose I.V. glucocorticoids in the acute inflammatory phase of the disease and surgical procedures in a burnt-out state. However, the results of the medical treatment are unsatisfactory, since up to 20% of patients are unresponsive and another 20% experience disease relapses after therapy withdrawal.

Therefore, as in other autoimmune diseases, new therapeutic options based on biological treatment are under experimental and clinical investigation. The efficacy of rituximab (RTX), has been reported since 2006. This humanized chimeric anti-CD-20 antibody blocks the activation and differentiation of B cells, since CD-20 protein is expressed on the surface of pre-B and mature B lymphocytes, but not on stem cells, pro-B lymphocytes and plasma cells. Therefore, treatment with RTX leads to specific elimination of B cells without affecting their regeneration or production of immunoglobulins by plasma cells.

Preliminary studies in patients with TAO indicate that blocking of CD-20 significantly and positively affected the clinical course of disease by rapid reduction of inflammation and degree of proptosis. The number of CD-20+ cells was lower in orbital tissues than in periphery.

RTX given locally as retrobulbar injections in patients resistant to glucocorticoid therapy resulted in a significant amelioration of the clinical symptoms. No side effects of that procedure were observed.

The outcome of the first randomized controlled study of rituximab or steroid treatment in TAO showed a superiority of RTX, used in single 500 mg dose, in the reduction of disease activity, improvement of QoL and prevention of relapse over the traditional methylprednisolone protocol. These effects do not seem to be related to TRab concentrations and may be due to the influence on antigen presentation (M. Salvi, ESE 2014).

RTX is the best tested therapeutic antibody in the treatment of TAO and may become an attractive treatment option.

